# PD-1/PD-L1 Checkpoint Inhibitors Are Active in the Chicken Embryo Model and Show Antitumor Efficacy *In Ovo*

**DOI:** 10.3390/cancers14133095

**Published:** 2022-06-23

**Authors:** Yan Wang, Xavier Rousset, Chloé Prunier, Paul Garcia, Emilien Dosda, Estelle Leplus, Jean Viallet

**Affiliations:** 1 R&D Department, Inovotion, 38700 La Tronche, France; xavier.rousset@inovotion.com (X.R.); chloe.prunier@inovotion.com (C.P.); paul.garcia@inovotion.com (P.G.); emilien.dosda@inovotion.com (E.D.); jean.viallet@inovotion.com (J.V.); 2 Université Grenoble Alpes, 38000 Grenoble, France; 3 Institute for Advanced Biosciences, Research Center Université Grenoble Alpes (UGA)/Inserm U 1209/CNRS 5309, 38700 La Tronche, France; 4 PDC*line Pharma, 38700 La Tronche, France; e.leplus@pdc-line-pharma.com

**Keywords:** chicken embryo, *in ovo*, CAM assay, PD-1/PD-L1 inhibitor, immuno-oncology

## Abstract

**Simple Summary:**

Cancer immunotherapy, also known as immuno-oncology (IO), has made impressive progress in recent decades and is becoming an essential approach for cancer treatments. For IO drug development, a pertinent preclinical model is indispensable for the rapid and efficient transition from preclinical evaluation through to clinical progress. To date, rodents represent the most-often used models for preclinical evaluation. However, their use presents several drawbacks, including ethical constraints, and time-consuming and costly experiments, which could slow down IO drug development. The aim of our study was to assess the use of the chicken embryo (*in ovo*) model as an alternative in vivo model for evaluating IO drugs. We confirmed *in ovo* the anti-tumor efficacy of programmed cell death protein-1 (PD-1)/programmed cell death-ligand 1 (PD-L1) checkpoint inhibitors based on the Chicken Chorioallantoic Membrane (CAM) assay, revealing the pertinence of the chicken embryo model in its use for IO research.

**Abstract:**

(1) Purpose: To assess the use of the chicken embryo (*in ovo*) model as an alternative *in vivo* model for immuno-oncology (IO) drug development, focusing on programmed cell death protein-1 (PD-1)/programmed cell death-ligand 1 (PD-L1) immune checkpoint inhibitors. (2) Methods: First, the presence of immune cells in the model was detected through the immunophenotyping of chicken peripheral blood mononuclear cells (PBMCs) based on fluorescence activated cell sorting (FACS) analysis and the immunohistochemistry (IHC) analysis of *in ovo* tumor-infiltrating lymphocytes. Second, the cross-reactivity between one anti-human PD-1 Ab, pembrolizumab (KEYTRUDA^®^), and chicken PD-1 was verified through the labelling of chicken splenocytes with pembrolizumab by FACS analysis. Third, the blockade effect of pembrolizumab on chicken PBMCs was assessed *in vitro* through cytotoxicity assay based on MTT. Fourth, the CAM assay was used to estimate the anti-tumor performance of pembrolizumab through the analyses of tumor growth and chicken immune cell infiltration in tumors. Finally, the efficacy of several PD-1 or PD-L1 inhibitors (nivolumab, atezolizumab and avelumab) on tumor growth was further assessed using the CAM assay. (3) Results: The presence of CD3^+^, CD4^+^, CD8^+^ T lymphocytes and monocytes was confirmed by FACS and IHC analyses. During *in vitro* assays, pembrolizumab cross-reacted with chicken lymphocytes and induced PD-1/PD-L1 blockade, which permitted the restoration of chicken T-cell’s cytotoxicity against human lung cancer H460 tumor cells. All these *in vitro* results were correlated with *in ovo* findings based on the CAM assay: pembrolizumab inhibited H460 tumor growth and induced evident chicken immune cell infiltration (with significant chicken CD45, CD3, CD4, CD8 and CD56 markers) in tumors. Furthermore, the potency of the CAM assay was not limited to the application of pembrolizumab. Nivolumab, atezolizumab and avelumab also led to tumor growth inhibition *in ovo*, on different tumor models. (4) Conclusions: The chicken embryo affords a physiological, immune reactive, *in vivo* environment for IO research, which allows observation of how the immune system defense against tumor cells, as well as the different immune tolerance mechanisms leading to tumor immune escape. The encouraging results obtained with PD-1/PD-L1 inhibitors in this study reveal the potential use of the chicken embryo model as an alternative, fast, and reliable *in vivo* model in the different fields of IO drug discovery.

## 1. Introduction

Cancer is the second leading cause of death worldwide behind cardiovascular disease, and the first cause of premature death in Europe [1]. Until lately, cancer treatment strategies mainly consisted of the surgical removal of the tumor, potentially combined with chemotherapy and/or radiotherapy. Although therapeutic solutions, along with the development of new approaches such as targeted therapies with tyrosine kinase inhibitors [2], have considerably improved patients’ overall survival during these past few decades, cancer-related mortality is still highly prevalent, with about 9 million deaths each year worldwide [3]. More recently, the immuno-oncology approach has emerged based on the works of James P. Allison and Tasuku Honjo [4,5,6,7]. This strategy focuses on the recruitment and reactivation of immune cells against tumor cells, offering new perspectives for patients with limited therapeutic options. Many current immuno-oncology studies focus on the use of immune checkpoint inhibitors, allowing the generation of an efficient antitumor immune response. Thus, the development of new immuno-oncology (IO) treatments could greatly improve the overall survival of cancer for patients with complex and advanced pathologies.

Because of the intricacy of the tumor microenvironment, a complex cellular ecosystem where many different immune cells interact with tumor cells [8], assessment of immunotherapies’ efficacy involves the use of *in vivo* models. Currently, rodents are the most-widely used animals in biological research. Besides their cost-effectiveness, most of their biological functions are well-described and relatively comparable to humans. However, rodent models are still far from optimal for IO applications, because these applications require a model system with a functionally intact immune system [9]. Current generations of rodent studies use humanized models, which greatly improve research and contribute to be more representative of the human organism. Rodents are immunodeficient to avoid transplant rejection. Inhibiting the immune system of the animal adds a serious bias in the studies, especially when trying to assess the efficacy of immunotherapies for example. Even though the latest generations of humanized rodent models (hu-BLT-SCID mice) can overcome this problem to some extent, with high levels of immune reconstitution, they still have great limitations, including an incomplete immune system and a high risk of developing Graft-versus-Host Disease (GvHD), as well as requiring costly and complex techniques [10,11,12].

During the first half of the 20th century, an alternative *in vivo* model has emerged: the chicken embryo [13,14]. Indeed, its Chorioallantoic Membrane (CAM) is a highly vascularized extraembryonic tissue that functions as a homolog of the mammalian placenta, and allows multiple applications in biomedical research [15,16,17,18,19,20,21]. Besides its various biological advantages such as its accessibility, cost-effectiveness, and reliability [22], the chicken embryo’s immune system develops gradually during its embryonic development. This allows the chicken to build its increasingly complex immune competency over time, which has been described as being biologically similar to humans’ [23].

In the early stages of embryonic development, very few immune cells can be observed within the model, which is itself considered to be immunodeficient before Embryonic Development Day (EDD) 10. This is because most of the embryo’s immune components have yet to mature [24]. This creates an ideal microenvironment, as it allows tumor cells to be xenografted onto the CAM with minimal risk of tumor rejection [25]. The embryonic immune system then becomes gradually more mature, until it is fully competent [26]. This allows us to perform an IO investigation in a physiological, immune-reactive, *in vivo* environment, for example from EDD10.5 for T-cells studies. Among the different IO drug classes, T-cell targeted immunomodulators, especially immune checkpoint inhibitors (e.g., anti-PD-1, PD-L1, CTLA4, LAG3), have seen the greatest increase in anticancer research in recent years. These immune checkpoints have also been identified in chickens with great similarity to humans’ [23]. Moreover, certain homology in terms of IO targets exists between humans and chickens makes the chicken embryo model particularly useful for IO investigations. For example, Shoichiro Horita et al., carried out the crystal structure study to clarify the mechanism of action of pembrolizumab, which is also one of the first FDA-approved PD-1 checkpoint inhibitors. Their findings revealed that the pembrolizumab epitope overlaps with PD-1 binding regions for PD-L1. Seven residues (Asn66, Gln75, Thr76, Asp77, Lys78, Ala132 and Glu136) of PD-1_ECD_ participate in polar interactions with both PemFv and PD-L1_ECD-N_. Therefore, the binding of pembrolizumab to PD-1 competes with the binding of PD-L1 to the receptor, and thus the presence of pembrolizumab could block the PD-1/PD-L1 signaling pathway [27]. Considering this work, a partial homology is found between humans and chicken embryos, *in situ* of pembrolizumab and PD-L1 competition residues. This observation leads us to think about the potency of the chicken embryo model for testing PD-1/PD-L1 inhibitors.

In this study, we first investigated the status of the chicken embryo’s immune system at a late development stage and checked *in vitro* the interaction between pembrolizumab and chicken immune T cells. We then validated the potency of the chicken embryo model as an *in vivo* IO testing model using clinically approved human PD-1/PD-L1 checkpoint inhibitors and showed the absence of toxicity of these IO agents *in ovo*.

## 2. Materials and Methods

### 2.1. Tumor Cell Culture

The human cancer cell lines NCI-H460, MDA-MB-231 and A375, were obtained from the American Type Culture Collection (ATCC). NCI-H460 cells were cultured in RPMI 1640 medium, supplemented with 10% fetal bovine serum (FBS), penicillin (100 units/mL)/streptomycin (100 μg/mL) and 1mM Pyruvate (Sigma-Aldrich, France). MDA-MB-231 and A375 cells were cultured in DMEM, supplemented with 10% fetal bovine serum (FBS) and penicillin (100 units/mL)/streptomycin (100 μg/mL) (Sigma-Aldrich, France). All cell lines were maintained at 37 °C in a 5% CO_2_ environment.

### 2.2. Immunophenotyping on Chicken Peripheral Blood Mononuclear Cells (PBMCs)

Chicken PBMCs were isolated from whole blood, freshly collected from chicken embryos at EDD16, by density-gradient centrifugation (Ficoll^®^ Paque Plus, Sigma-Aldrich, France). Cells were then collected using Fluorescence-Activated Cell Sorter (FACS) tubes, washed and resuspended in 100 µL staining buffer (Phosphate-Buffered Saline (PBS) with 2% FBS). The following antibodies were used for staining: anti-chicken CD45-FITC (Thermo Fisher Scientific, France), anti-Chicken CD3 (CT-3)-Pacific Blue^®^ (CliniSciences, France), anti-chicken CD8-PE (Thermo Fisher Scientific, France), anti-chicken CD4-PE (Thermo Fisher Scientific, France), anti-chicken KUL01-PE (Thermo Fisher Scientific, France). 7-Aminoactinomycin D (7-AAD) (Thermo Fisher Scientific, France) was used to check the cell viability.

All data were acquired with a BD FACSCanto II flow cytometer using BD FACSDiva software (BD Biosciences, France) and analyzed on BD FACSuite software (BD Biosciences, France).

### 2.3. Immunohistochemistry (IHC) Detection of Tumor-Infiltrated Lymphocytes

H460 tumors grown on CAM were collected at EDD18, washed in PBS, and fixed in 4% paraformaldehyde for 48 h at 4 °C. The tumors were then cleaned of any remaining CAM tissue, trimmed, and embedded in paraffin cassettes. IHC staining was performed on 4 µm FFPE sections using the Leica Bond max system (Leica Biosystems Newcastle Ltd., UK). Slides were baked for 30 min at 60 °C, dewaxed and pretreated with an epitope-retrieval solution: CD3 (Abcam, France), CD8 (Biorbyt, France) and CD4 (Abcam, France). Detection was performed using the Leica Bond Polymer Refine HRP kit (Leica Biosystems Newcastle Ltd., UK). All slides were counter-stained with Hematoxylin. Illustrative pictures were acquired with a Leica CTR 6500 confocal microscope (Leica Microsystems, Germany) at 20× magnification.

### 2.4. Binding of Pembrolizumab to Chicken Immune Cells

Chicken PBMCs were purified as mentioned previously (Section 2.2). Chicken spleens were collected at EDD16, and single-cell suspensions of the spleens were prepared using the mechanical dissociation method. Both PBMCs and splenocytes were resuspended at 1 × 10^6^ cells/mL in RMPI, supplemented with 10% FBS, 2 mM L-glutamine, penicillin (100 units/mL)/streptomycin (100 μg/mL) in a 6-well plate pre-coated with anti-Chicken CD3 (CT-3) (CliniSciences, France) at 1 µg/mL. Recombinant Chicken IL-2 (CliniSciences, France) was added to the culture as well, at 10 ng/mL. As reference, human PBMCs purified from a healthy donor (Établissement Français du Sang, Grenoble, France) were cultured under the same conditions, with anti-human CD3 (OKT3) (Thermo Fisher Scientific, France) at 1 µg/mL and recombinant human IL-2 (Thermo Fisher Scientific, France) at 10 ng/mL. Cells were maintained in a humidified incubator at 37 °C and 5% CO_2_ for 6 days, cytokines were renewed every three days.

For FACS analysis, pembrolizumab (KEYTRUDA^®^, MSD, France) and isotype human IgG4 Kappa (Bio-Rad, France) were labelled with the far-red fluorescent dye CF633 (Sigma-Aldrich, France), according to the manufacturer’s instructions. Precultured chicken PBMCs and splenocytes were then stained with anti-chicken CD45-FITC (Thermo Fisher Scientific, France) and pembrolizumab-CF633 (or isotype human IgG4-CF633).

All data were acquired with a BD LSR II flow cytometer using BD FACSDiva software (BD Biosciences, France) and analyzed on FCS Express 7 software (De Novo Software).

### 2.5. In Vitro Pembrolizumab Functionality Test

PBMCs were purified as previously mentioned (Section 2.2) and were resuspended at 1.10^6^ cells/mL in RPMI supplemented with 10% FBS, 2 mM L-glutamine, penicillin (100 units/mL)/streptomycin (100 μg/mL). Phytohemagglutinin-L (PHA-L) (Sigma-Aldrich, France) was added at 5 µg/mL to the medium and the cell culture was maintained at 37 °C, 5% CO_2_ for 72 h. During the last 24 h of stimulation with PHA-L, pembrolizumab (KEYTRUDA^®^, MSD, France) was added to the cell culture at 10 µg/mL. PHA-L stimulated PBMCs, with (or without) pembrolizumab treatment, were then collected, washed by PBS and resuspended in co-culture with H460 tumor cells at different ratios (10:1, 20:1 and 40:1), respectively, for 4 h. Cultures with only H460 cells and PBMCs were applied as controls. Lastly, the viability of H460 cells was measured by the MTT-based *in vitro* cytotoxicity test (Sigma-Aldrich, France), for which the quantity of formazan was measured by recording changes in absorbance at 570 nm, with a correction at 630 nm. The tumor cells’ viability was calculated using the formula below:

% Tumor cells viability = 100 × (absorbance^sample (tumor cells + PBMCs)^ − absorbance^control_only PBMCs^)/absorbance^control_only tumor cells^

### 2.6. Western Blot Analysis of PD-L1 Expression In Ovo

H460 tumors grown on CAM were collected at EDD16, washed in PBS, and quickly cleaned of any remaining CAM tissue. Tumors were then ground using a sterile pestle in a RIPA lysis buffer (Thermo Fisher Scientific, France) containing protease inhibitors (Roche, France). The resulting suspension was centrifuged at 10,000× *g* (4 °C) for 20 min. The supernatant was transferred to a clean 1.5 mL tube and its protein concentration was evaluated using the Pierce™ BCA protein assay kit (Thermo Fisher Scientific, France).

Western blot analysis was performed by Synthelis SAS (La Tronche, France). We subjected 30 μg micrograms of cellular proteins to electrophoresis on 4–12% Bis-Tris gel (Thermo Fisher Scientific, France). Transfer to the nitrocellulose membrane was performed using Trans Blot Turbo (Bio-Rad, France). Membranes were saturated in 5% BSA TBS Tween 0.05% and incubated with a primary antibody (Rabbit anti-PD-L1, Cell Signaling Technology, France; Rabbit anti-GAPDH, Cell Signaling Technology, France) for 1 h. Membranes were then washed in PBS and incubated with a secondary antibody (anti-Rabbit IgG, HRP-linked, Cell Signaling Technology, France) for 1 h. Signal revelation was performed using ECL on gelDoc system (Bio-Rad, France). Membranes were exposed for 180 s for PD-L1 and for 16 s for GAPDH.

### 2.7. Chicken Chorioallantoic Membrane Assay (CAM) Assay

Fertilized white leghorn chicken eggs were obtained from Couvoir Hubert, France. Eggs were incubated at 37.5 °C with 50% relative humidity for 9 days. At EDD9, the CAM was dropped down by drilling a small hole through the eggshell into the air sac and a 1 cm^2^ window was cut in the eggshell above the CAM. Tumor cells were detached with trypsin, washed with complete medium and suspended in graft medium. Then, an inoculum of 1.10^6^ cells was added onto the CAM.

At EDD10, tumors began to be detectable. Treatments with pembrolizumab (KEYTRUDA^®^, MSD, France), nivolumab (OPDIVO^®,^ BMS, France), were performed 5 times at EDD10, 12, 13, 15 and 17; pembrolizumab was tested at 1, 1.5 and 2 mg/kg; and nivolumab was tested at 2 mg/kg. The treatments with atezolizumab (TECENTRIQ^®^, Roche, France) and avelumab (BAVENCIO^®^, Merck, France) were performed 4 times at EDD10, 12, 14 and 16; atezolizumab was tested at 2 mg/kg; and avelumab was tested at 2, 4 and 8 mg/kg. For all PD-1 and PD-L1 inhibitors tests, the vehicle (PBS) was administrated in parallel as the control.

Embryonic viability was checked daily. The CAM assay was ended at EDD18, and the upper portion of the CAM (with tumor) (*n* = 10–20/group) was removed, washed with a PBS buffer and then directly transferred into 4% paraformaldehyde (fixation for 48 h at 4 °C). Tumors were then carefully cut away from the normal CAM tissue and weighed for a quantitative evaluation of tumor growth. The number of dead embryos was also counted at EDD18, to evaluate treatment-induced embryo toxicity. The final death ratio and a Kaplan-Meyer curve were reported for all groups.

### 2.8. Quantitative Evaluation of Immune Cells Infiltration by RT-qPCR

Among the tumors collected at EDD18, 6 tumors per group were used to evaluate the infiltration of immune cells. Each tumor sample was cut to a small size (<0.5 cm^3^) and kept in 5 volumes of RNA Safeguard (Dutscher, France) solution at 4 °C. On the next day, tumor samples were either frozen at –80 °C or used directly for RNA extraction (MagMAX mirVana Total RNA Isolation kit, Thermo Fisher Scientific, used in Thermo Fisher Scientific automated machine KingFisher Duo Prime). Extracted RNA was analyzed by RT-qPCR with specific primers for chicken CD45, CD3, CD8, CD4 and CD56 sequences (PrimePCR™ Probe Assay and iQ Multiplex Powermix, Bio-Rad, France). For all points measured via qPCR, the corresponding expression of human GAPDH (PrimePCR™ Probe Assay, Bio-Rad, France) was also analyzed, as the reference gene expression, and used to normalize immune biomarker expression between tumor samples. Each sample’s Cq, mean Cq and relative amounts of immune cells for each group were directly calculated and managed by the Bio-Rad^®^ CFX Maestro software (Bio-Rad, France).

### 2.9. Statistical Analysis and Significance

All quantitative data were analyzed with the specialized computer software Prism^®^ (GraphPad Software). For comparison between two groups, an unpaired *t*-test was applied. For comparison between more than two groups, a one-way ANOVA analysis (with post-tests between each couple of groups) was performed. For all analyses, the statistical difference between groups is indicated on graphs with stars: No stars, no statistical difference (*p*-value > 0.05); one star (*), 0.05 ≥ *p*-value > 0.01; two stars (**), 0.01 ≥ *p*-value > 0.001; three stars (***), 0.001 ≥ *p*-value > 0.0001; four stars (****), 0.0001 ≥ *p*-value.

## 3. Results

### 3.1. Immunophenotypic Characterization of Chicken Peripheral Blood Mononuclear Cells

**Figure 1:**
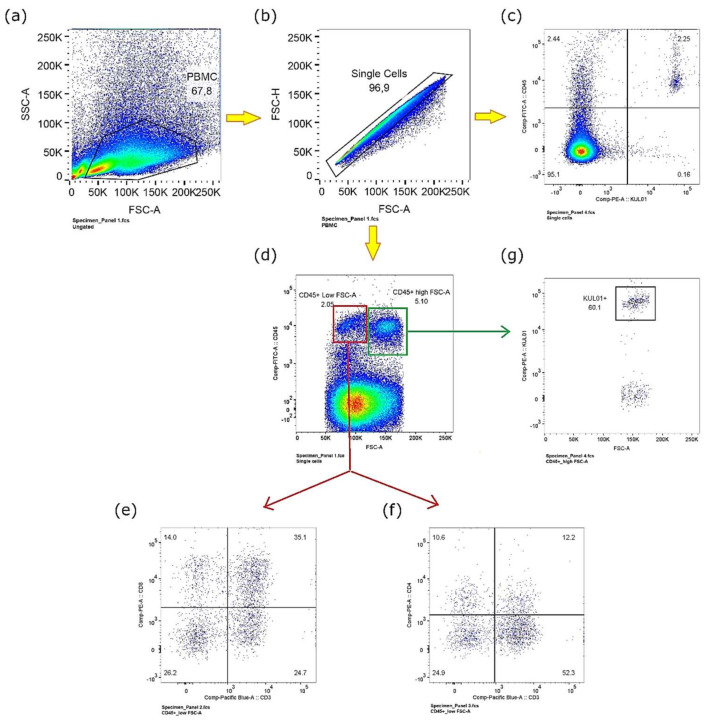
Immunophenotyping on Chicken Peripheral Blood Mononuclear Cells: (**a**) PBMCs were gated based on FSC and SSC; (**b**) Singlet cells were selected from gated PBMCs; (**c**) Monocytes were identified within singlet gated PBMCs through labeling with fluorochrome-conjugated anti-chicken CD45 and anti-chicken KUL01 antibodies; (**d**) Two cell subsets were observed with CD45^+^ cells, FSC-low or FSC-high; (**e**) CD8^+^ lymphocytes and (**f**) CD4^+^ lymphocytes were identified with CD45^+^/FSC-low population; (**g**) Monocytes clustered more in CD45^+^/FSC-high population.

Chicken PBMCs were collected and purified from chicken embryos at EDD16 (Figure 1), presenting a good viability (>99% alive cells) detected by 7AAD staining (plots data not shown). Based on these samples, three major immune cell subsets, CD8^+^ T lymphocytes (CD45^+^/CD3^+^/CD8^+^), CD4^+^ T lymphocytes (CD45^+^/CD3^+^/CD4^+^) and monocytes (CD45^+^/KUL01), were characterized. Monocytes were easily identified in single PBMCs (Figure 1b) through staining with fluorochrome-conjugated anti-chicken CD45 and anti-chicken KUL01 antibodies (Figure 1c). In parallel, the tracking of CD8^+^ and CD4^+^ T lymphocytes was first attempted within CD45^+^ singlet cells, whereas the visualization of both T cell lineages was not evident. It was interesting to find that CD45^+^ PBMCs have two principal phenotypes, either CD45^+^/Low-FSC-A or CD45^+^/High-FSC-A (Figure 1d). Therefore, we further explored CD8^+^ and CD4^+^ T lymphocytes in these subsets, respectively. In this way, CD8^+^ and CD4^+^ T lymphocytes were finally sought out in the CD45^+^/Low-FSC sub-population (Figure 1e,f). The proportion of CD8^+^ lymphocytes in PBMCs is higher than CD4^+^ lymphocytes. We also noticed that monocytes were more clustered in the CD45^+^/High-FSC sub-population (Figure 1g).

### 3.2. Tumor-Infiltrating Lymphocytes Are Visualized In Ovo

**Figure 2:**
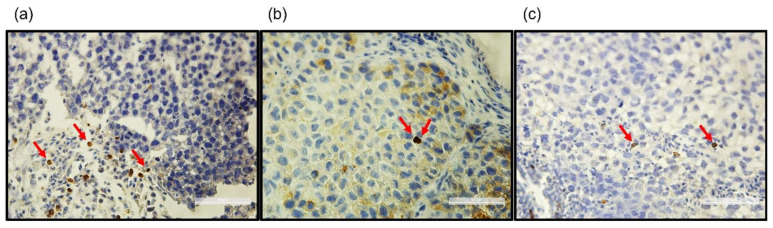
Representative examples of the immunohistochemical staining of the lymphocyte markersCD3 (**a**), CD4 (**b**) and CD8 (**c**), pointed by red arrow, for H460 tumor xenografted *in ovo*. Bar = 50 µm.

The presence of chicken embryonic immune cells was also confirmed by the IHC analysis of tumors xenografted on CAM. As shown in Figure 2, the presence of CD3^+^, CD4^+^ and CD8^+^ immune cell populations was detected within the tumor. As observed in the tumor samples, these tumor-infiltrated immune cells illustrate the existence of the tumor microenvironment.

### 3.3. Anti-Human PD-1 Cross-Reacts with Activated Chicken Splenocytes In Vitro

**Figure 3:**
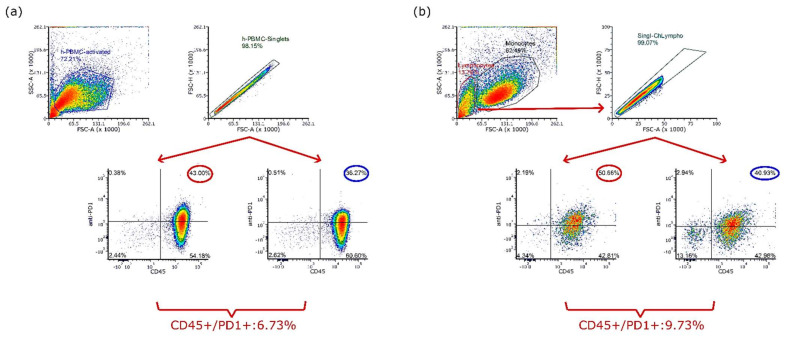
Pembrolizumab binding assay measured by flow cytometry. Before the FACS analysis, the chicken splenic monocytes and human PBMCs were pre-activated using anti-CD3 Ab at 1 µg/mL and IL2 at 10 ng/mL (chicken or human species), respectively, for 6 days. The cells were then labelled with fluorochrome-conjugated anti-CD45 and fluorochrome-conjugated pembrolizumab for FACS analysis. One human IgG4 Kappa isotype control Ab was also applied for excluding the non-specific binding: (**a**) By subtracting non-specific binding revealed by isotype control, 6.73% of activated human PBMCs were CD45^+^/PD-1^+^; and (**b**) 9.73% of inactivated chicken splenic lymphocytes were CD45^+^/PD-1^+^.

To estimate the cross-activity between anti-human PD-1 Ab and chicken PD-1, the binding capacity of pembrolizumab to chicken immune cells was estimated through FACS analysis. As PD-1 is rarely expressed by naive T cells, chicken splenocytes were first stimulated by anti-chicken CD3 and recombinant chicken IL-2. In parallel, human PBMCs were stimulated with anti-human CD3 and recombinant human IL-2, serving as the reference. After 6 days of pre-activation, cells were characterized through fluorochrome-conjugated anti-CD45 and fluorochrome-conjugated pembrolizumab. One human IgG4 Kappa isotype control was used to exclude non-specific binding. Related FACS analysis results are shown in Figure 3. For both human and chicken samples, we noted a non-specific binding revealed by the isotype IgG4 Ab. To interpret the specific pembrolizumab binding, the non-specific binding revealed by isotype IgG4 was subtracted from the pembrolizumab binding data. Thus, we observed 6.73% of CD45^+^/PD-1^+^ cells in activated human PBMCs (Figure 3a) and 9.73% of CD45^+^/PD-1^+^ cells in lymphocyte subsets of activated chicken splenocytes (Figure 3b).

### 3.4. Pembrolizumab Induces PD-1/PD-L1 Blockade and Restores Chicken T-Cell’s Cytotoxicity against Tumor Cells

**Figure 4:**
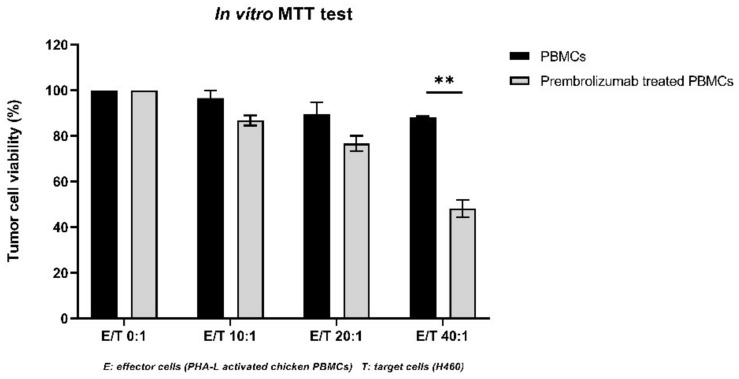
Restored effector cytotoxicity of PHA-L activated chicken PBMCs following pre-treatment with pembrolizumab was revealed through an MTT-based assay. Purified chicken PBMCs were activated *in vitro* by incubation in PHA-L at 5 µg/mL for 72 h and then treated with pembrolizumab at 10 µg/mL for 24 h Cytotoxicity of PHA-L stimulated PBMCs was then estimated using a co-culture with H460 cells as targets, at different E/T ratios (10:1, 20:1 and 40:1). PHA-L activated PBMCs without pembrolizumab pre-treatment were applied as the control. Tumor cell viability was measured through an MTT-based assay. The restored cytotoxicity of effector cells related to the pembrolizumab pre-treatment was significant when the E/T ratio was 40/1, *p* = 0.0089 (**).

Because pembrolizumab binds to chicken immune cells, the effect of this binding on effector cells was further evaluated through an *in vitro* cytotoxicity assay. After the *in vitro* stimulation with PHA-L, activated chicken PBMCs were pre-treated with or without pembrolizumab. Effector cells (activated chicken PBMCs) were then incubated with target tumor cells (H460) at different ratios (10:1, 20:1 or 40:1). The effector cells’ cytotoxicity was interpreted using target tumor cells’ viability, which was measured based on an MTT assay. In Figure 4, we found that the target cells’ viability declined with the increased ratio between effector and target cells, only when effector cells were pre-treated with pembrolizumab. In contrast, if effector cells were not pre-treated with pembrolizumab, the viability of target cells was not evidently impacted even if more effector cells were added. These results revealed that the pre-treatment of activated chicken PBMCs with pembrolizumab can induce the blockage of PD-1 and PD-L1 interaction, which contributed to the reinvigoration of T cell’s effector functions against tumor cells. When compared to the PBMCs^PHA-L^ without pembrolizumab treatment, the restored cytotoxicity of PBMCs^PHA-L^ after the pre-treatment with pembrolizumab was significantly observed when the Effector/Target (E/T) cells ratio was 40:1 (i.e., 51.82% mortality of target tumor cells, *p* = 0.0089).

### 3.5. PD-L1 Epitope Is Preserved on Tumors Grown on the CAM

**Figure 5:**
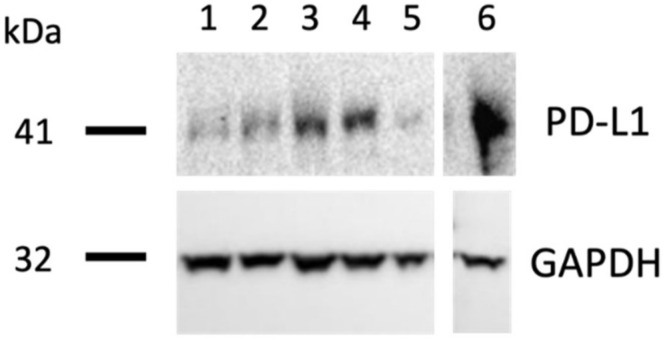
PD-L1 expression level on *in ovo* xenografted H460 tumors. Representative Western blot of H460 tumors collected at EDD16. Lane 1: tumor 1, lane 2: tumor 2, lane 3: tumor 3, lane 4: tumor 4, lane 5: tumor 5, and lane 6: positive control. Human GAPDH was used as the internal control. Samples were loaded on the same membrane, but lanes were rearranged for the purposes of the figure. The original uncropped WB image is presented in Supplementary Figure S1.

One concern for any *in vivo* IO study is whether the immune epitope expression on tumor cells can be preserved after xenografting. To address this question, we checked the expression level of PD-L1 on H460 tumors xenografted onto the CAM through Western blot analysis. At EDD16, 7 days after *in ovo* xenografting, PD-L1 expression in H640 tumors is detectable, and the Western blot revelation is shown in Figure 5.

### 3.6. Pembrolizumab Induces H460 Lung Tumor Growth Regression In Ovo at Safe Doses

**Figure 6:**
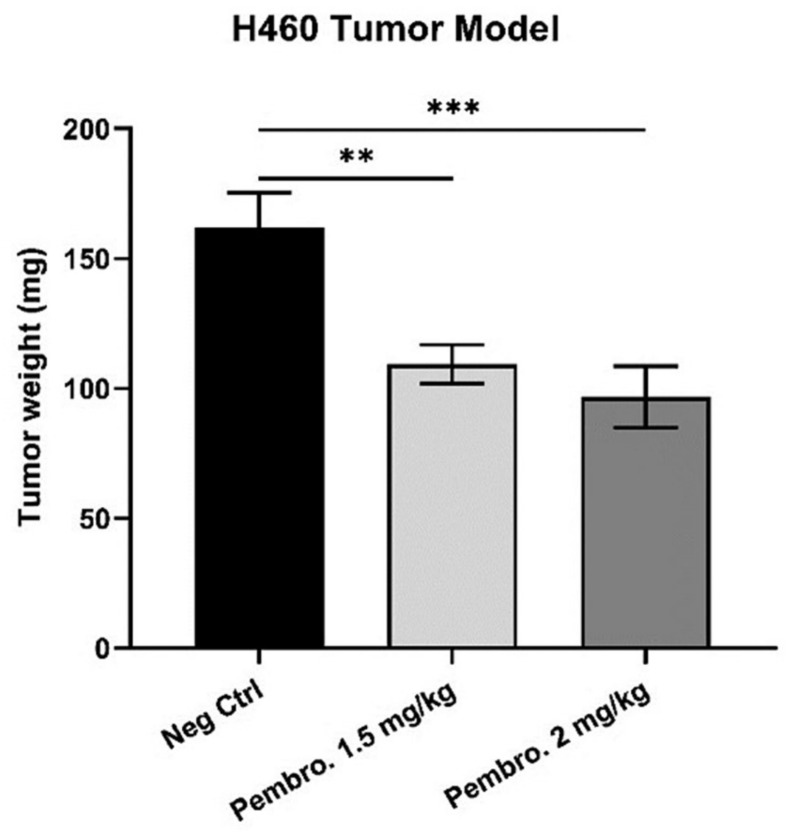
Tumor weights (mean values ± SEM) of *in ovo* xenografted H460 tumors after treatments with pembrolizumab. Pembro. 1.5 mg/kg vs. Neg Ctrl, *p* = 0.0065 (**); Pembro. 2.0 mg/kg vs. Neg Ctrl, *p* = 0.0007 (***).

*In ovo*, the effect of pembrolizumab was first evaluated based on the tumor growth readout. Pembrolizumab was tested at 1.5 and 2 mg/kg on the H460 tumor model. The tumor growth inhibition induced by pembrolizumab is presented in Figure 6. We noticed that pembrolizumab led to a significant tumor growth inhibition on the H460 model at both tested doses: 32.53% of tumor growth regression at 1.5 mg/kg, *p* = 0.0065; 40.27% of tumor growth regression at 2.0 mg/kg, *p* = 0.0007.

**Figure 7:**
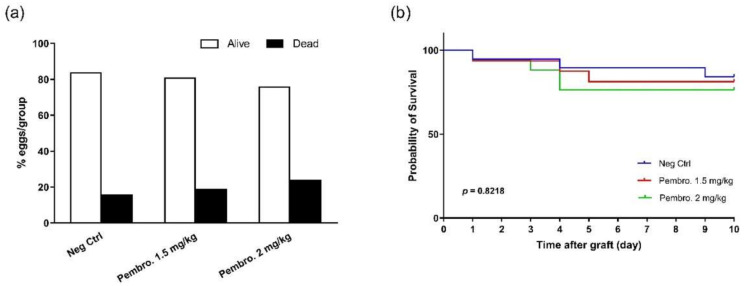
Survival of H460 Xenografted Embryos Following Pembrolizumab Treatments. (**a**) The final alive and dead ratios were recorded; (**b**) Kaplan–Meyer curve presents the survival evolution after *in ovo* H460 xenografting at EDD9.

Other than for anti-tumor efficacy, the *in vivo* toxicity of pembrolizumab was also estimated, and the results are presented in Figure 7. Embryonic viability was checked daily, and the number of dead embryos was also counted at EDD18. The final alive and dead ratios were recorded in Figure 7a. The Kaplan–Meyer curve (Figure 7b) presents in greater detail the survival evolution during *in ovo* experimentation.

### 3.7. Pembrolizumab Increases Immune Cell Infiltration in H460 Tumor Xenografted In Ovo

**Figure 8:**
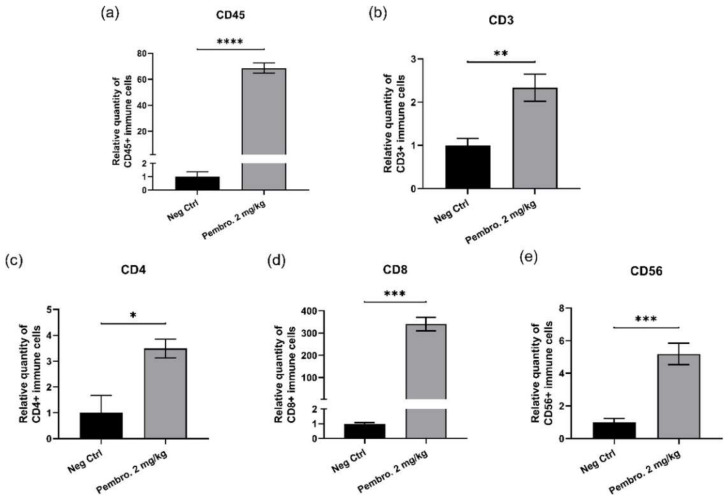
Relative mRNA expression levels of chicken immune markers (mean values ± SEM) in *in ovo* xenografted H460 tumors after treatments with pembrolizumab. Significant up-regulation was found for all detected genes: (**a**) CD45, up-regulated 68.5 times (*p* < 0.0001, ****); (**b**) CD3, up-regulated 2.3 times (*p* = 0.0062, **); (**c**) CD4, up-regulated 3.5 times (*p* = 0.0124, *); (**d**) CD8, up-regulated 340 times (*p* = 0.0001, ***); (**e**) CD56, up-regulated 5.2 times (*p* = 0.0008, ***).

To validate the mechanism of action of pembrolizumab *in ovo*, the pembrolizumab performance was further studied for the immune infiltration of tumors. After the treatments with pembrolizumab, *in ovo* xenografted H460 tumors were collected at EDD18 and total RNAs were extracted from tumor samples and transcribed to cDNAs for qPCR analysis. The mRNA expression of different immune markers: CD45, CD3, CD4, CD8 and CD56, were determined. Results are presented in Figure 8. When compared to the Negative Control, significant up-regulation was found for all analyzed immune markers: CD45, up-regulated 68.5 times (*p* < 0.0001); CD3, up-regulated 2.3 times (*p* = 0.0062); CD4, up-regulated 3.5 times (*p* = 0.0124); CD8, up-regulated 340 times (*p* = 0.0001); CD56, up-regulated 5.2 times (*p* = 0.0008).

### 3.8. Efficacy of PD-1 Checkpoint Inhibitors Are also Confirmed with MDA-MB-231 Triple-Negative Breast Cancer In Ovo

**Figure 9:**
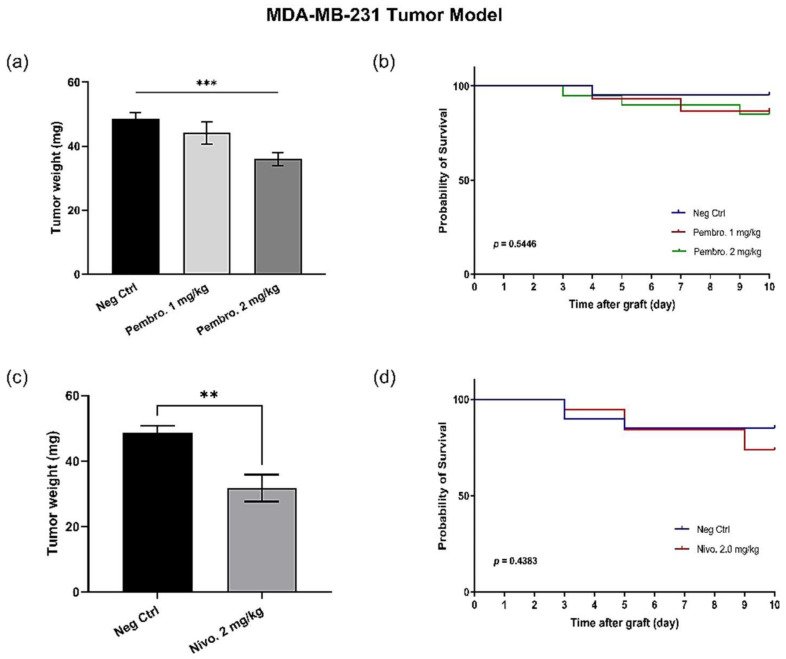
*In ovo* PD-1 inhibitors’ treatment in the MDA-MB-231 tumor model, (**a**) Pembrolizumab induced 8.87% of tumor growth inhibition at 1.0 mg/kg (*p* = 0.444) and 25.8% of regression at 2 mg/kg (*p* = 0.0007, ***); (**b**) No evident toxicity was induced by pembrolizumab treatment; (**c**) Nivolumab led to 34.77% of tumor growth regression (*p* = 0.0031, **); (**d**) No evident toxicity was induced by nivolumab treatment.

Beyond the H460 tumor model, pembrolizumab has also been tested with the human breast cancer MDA-MB-231. Pembrolizumab was tested at two doses, 1.0 mg/kg and 2.0 mg/kg. On the MDA-MB-231 model, the tumor growth diminution was 8.87% at 1.0 mg/kg (*p* = 0.444) and a significant tumor growth regression was observed, at 2 mg/kg (25.8% of regression, *p* = 0.0007) (Figure 9a), and no evident toxicity was observed at any dose (Figure 9b). Moreover, another anti-human PD-1 inhibitor (nivolumab) has been tested *in ovo* and induced significant tumor growth inhibition at 2 mg/kg on this tumor model (*p* = 0.0031) (Figure 9c), without leading to evident mortality (Figure 9d).

### 3.9. PD-L1 Checkpoint Inhibitors Are Efficient In Ovo

**Figure 10:**
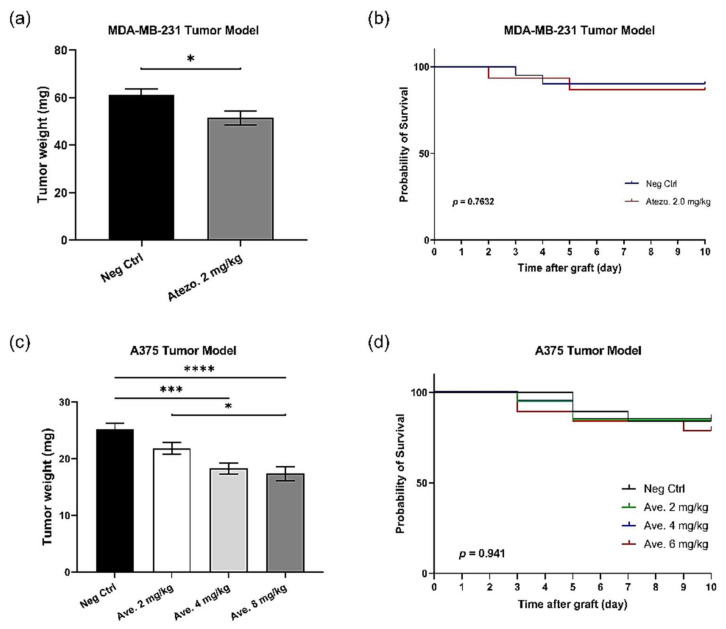
*In ovo* PD-L1 inhibitors’ treatment in the MDA-MB-231 tumor model and the A375 tumor model: (**a**) On the MDA-MB-231 tumor model, atezolizumab at 2 mg/kg induced 15.97% of tumor regression (*p* = 0.0146, *); (**b**) In the MDA-MB-231 tumor model, no evident toxicity was induced by atezolizumab treatment; (**c**) Iin the A375 tumor model, avelumab led to 13.41% tumor regression at 2 mg/kg (*p* = 0.1217), 27.4% tumor regression at 4 mg/kg (*p* = 0.001, ***), and 31.0% tumor regression at 8 mg/kg (*p* < 0.0001, ****). The statistical difference between 2 and 8 mg/kg was *p* = 0.026 (*), (**d**) In the A375 tumor model, no evident toxicity was induced by avelumab treatment.

The PD-1/PD-L1 pathway could be interfered with, through the blockade of either ligand. Thus, two PD-L1 inhibitors (atezolizumab and avelumab) were also tested on different tumor models in this study. As shown in Figure 10, atezolizumab at 2 mg/kg resulted in a regression of 15.97% on MDA-MB-231 (*p* = 0.0146) (Figure 10a). For the second anti-human PD-L1, avelumab, its tumor growth inhibition effect was dose-dependent, and the tumor growth regression at 2 mg/kg was 13.41% (*p* = 0.1217), whereas the evident tumor regression was obtained at 4 mg/kg (27.4%, *p* = 0.001), and 8 mg/kg (31.0%, *p* < 0.001); the tumor regression was significantly reinforced when the dose was increased from 2 to 8 mg/kg (*p* = 0.026) (Figure 10c). For both PD-L1 inhibitors, no significant toxicity was observed at any dose (Figure 10b,d).

## 4. Discussion

Cancer immunotherapy, also known as immuno-oncology, is emerging as an essential approach for cancer treatments. Efficient and successful IO drug development requires adequate investment and appropriate tools to facilitate the rapid transition from preclinical evaluation through to clinical development. These tools include relevant preclinical models, pertinent biomarkers for clinical prediction and monitoring, and evolving clinical trial designs that allow rapid and efficient evaluation during the drug development process [28]. Although rodents represent the most-often used models for preclinical evaluations, their use includes many drawbacks, including ethical restraints, time-consumption, and costly experimentation, which slow down IO drug development. For this reason, we wanted to validate the use of an alternate model: The chicken embryo, which is more efficient, faster, and is 3R-compliant and less expensive than rodents for IO drug discovery.

Indeed, the immune status of the chicken embryo model for IO testing needs to be clarified, including the immune checkpoints’ involvement in tumor growth *in ovo*. To address these concerns, we first investigated different components in the embryonic immune system. It is widely accepted that T cells play a central role in the fight against cancer. Among the different T cell subsets, CD8^+^ cytotoxic T cells are key effector cells in anti-tumor immunity [29,30]. In parallel, another T cell population, CD4^+^ helper T cells, is indispensable for the generation and maintenance of effective CD8^+^ cytotoxic and memory T cells. Its helping function facilitates optimal expansion, trafficking, and effector function of CD8^+^ T cells, thereby contributing to tumor protection [31,32,33]. Furthermore, a specialized subset of CD4^+^ T cells, CD4^+^CD25^+^ regulatory T cells (TRegs), effectively hampers anti-tumor immune responses, and this has been proposed as one of the major tumor immune evasion mechanisms [33,34]. In addition, monocytes, the largest type of white blood cells, play an important role in the adaptive immunity process. Monocytes and monocyte-derived cells, including macrophages and dendritic cells, also direct T cell activation and function via cues that range from being immunosuppressive to immunostimulatory, and are therefore considered to be important regulators of cancer development and progression [35]. Thus, the immunophenotyping of chicken embryo PBMCs was focused on these immune cell subsets. As shown in Figure 1, PMBCs freshly purified at EDD16, were first gated in terms of morphology (FSC/SSC) and then singlet cells were targeted for further analysis. Within CD45^+^ cells, monocytes were easily visualized by KUL1 labelling (2.25% CD45^+^/KUL1^+^ cells in gated single cells). However, it was not easy to target CD45^+^/CD3^+^/CD8^+^ and CD45^+^/CD3^+^/CD4^+^ cells directly within gated single cells (data not shown). Meanwhile, we noticed two sub-populations with different FSC morphologies within CD45^+^ single cells (CD45^+^/FSC^low^ and CD45^+^/FSC^high^). Thus, we continued our exploration in these two subpopulations, respectively. It was interesting to observe the CD3^+^/CD8^+^ and CD3^+^/CD4^+^ immune cells in the CD45^+^/FSC^low^ population (35.1% of CD3^+^/CD8^+^ cells, 12.2% of CD3^+^/CD4^+^ cells), while KUL1^+^ cells were more enriched in the CD45^+^/FSC^high^ population (60.1% of KUL1^+^ cells). These phenotype characteristics are similar to humans’ and rodents’. However, chicken PBMCs possess more CD8^+^ lymphocytes than CD4^+^ lymphocytes, which is different from human and murine species. Thereby, three distinct immune cell populations, CD8^+^ T cells, CD4^+^ T cells and monocytes, were well characterized in the chicken embryo. The CD8^+^ and CD4^+^ T cells issued from chicken embryos have been visualized as well through IHC analysis on tumors xenografted on the CAM (Figure 2). This not only confirmed the existence of effector immune cells in the chicken embryo model, but also demonstrated the infiltrating capacity of these immune cells, illustrating the rich tumor microenvironment in xenografts on CAM. CD8^+^ T cells, CD4^+^ T cells and monocytes are relevant cell subsets ensuring pembrolizumab efficacy, while there are still other important immune cell subsets that need to be analyzed if for other IO agents’ application *in ovo*. For example, natural killer (NK) cells for ADCC agents testing; B cells for immunoglobulins enhancement, etc.

In this study, we focused on the PD-1/PD-L1 pathway for validating the potential use of the chicken embryo model for IO development, because research on immune checkpoint inhibitors during the past decade has expanded exponentially and has improved treatments for a broad spectrum of cancers [36]. When PD-L1 is overexpressed on the surface of malignant tumor cells and binds to PD-1, the proliferation of PD-1^+^ effector cells could be inhibited, leading to immune escape of tumors and therefore treatment failure. The blockade of PD-1 or PD-L1 with specific antibodies can enhance T cell responses and mediate antitumor activity [37]. Pembrolizumab was one of the first FDA-approved antibodies blocking the PD-1 checkpoint. Shoichiro Horita et al. carried out the crystal structure study for identifying the residues in human PD-1 recognized by pembrolizumab and that interact with PD-L1. Their findings revealed that the binding of pembrolizumab to PD-1 would compete with the binding of PD-L1 to the receptor and thus the presence of pembrolizumab could block the PD-1/PD-L1 signaling pathway [27]. Considering this work, we noticed a certain encouraging homology between humans and chicken embryos, in terms of the pembrolizumab and PD-L1 competition region. Thus, we first addressed *in vitro* the cross-activity between anti-human PD-1 (pembrolizumab) and chicken PD-1. In fact, PD-1 expression is absent or low on the surface of resting immune cells but can be induced in different immune populations such as CD4^+^ and CD8^+^ T cells, NK cells, B cells, macrophages, and some dendritic cell (DC) subsets [38,39,40]. The use of anti-CD3 and IL2 for human T cell activation has been reported for many years [41,42], as well as in the mouse model [43]. As shown in Figure 3, for both types of samples, we noticed a non-specific binding revealed by the isotype IgG4 Ab. This unwanted binding frequently occurs between the Fc fragment of antibodies to Fc receptor-expressing cells such as B cells, monocytes, as well as T-cell lineage within a narrow window following cellular activation. This problem could be resolved via FcR blocking before FACS Ab labelling. We did not perform FcR blocking before FACS analysis because the FcR in the chicken has not been clarified and therefore no chicken FcR blocking reagent is available on the market. To address this issue, we used an isotype IgG4 control to anticipate the non-specific binding, for both human and chicken samples. Therefore, the percentage of CD45^+^/PD-1^+^ cells detected in pembrolizumab labelling tubes was subtracted by the non-specific binding revealed by isotype IgG4. Thus, we observed 6.73% of CD45^+^/PD-1^+^ cells in activated human PBMCs and 9.73% of CD45^+^/PD-1^+^ cells in lymphocyte subsets in activated chicken splenocytes. The percentage of CD45^+^/PD-1^+^ cells (6.73%) observed in activated human PBMC samples was slightly lower than that reported by Sunao Sugita et al., with anti-CD3 treated Jurkat cells (17%) and anti-CD3 treated CD4+ T cells (8%) [44]. The possible reasons for this difference are: (1) the pre-stimulated samples contained mixed cell populations and thus displayed different behavior from pure cell lines; (2) the pre-stimulation time with anti-CD3 and IL2 (6 days) in this study was long, and T cell exhaustion occurred. We performed 6 days of pre-stimulation to induce T cell activation and T cell proliferation. However, most studies focusing on PD-1 upregulation following anti-CD3 activation checked the PD-1 expression 48–78 h after stimulation. It is possible that the PD-1 upregulation peak induced by anti-CD3 had passed before day 6 and the stimulation with only anti-CD3 and IL2 was insufficient for sustaining PD-1 expression. In addition to the chicken splenocytes, we also attempted the pembrolizumab binding assay with chicken PBMC samples, from which we did not observe evident CD45^+^/PD-1^+^ cells (data not shown). The probable reason for the failure with chicken PBMCs samples was the low cell viability, observed after 6 days of pre-stimulation (about 30% of viable cells).

With these findings, we wondered whether T-cell effector functions can be revived by this anti-PD-1 antibody blocking. We applied an alternative, PHA-based, *in vitro* stimulation protocol on chicken embryo PBMCs, which permitted a rapid activation of T cells with increased PD-1 expression [45]. Activated PBMCs were then treated with (or without) pembrolizumab, and then co-cultured with the target H460 tumor cells. We found that the viability of the targeted H640 cells was evidently reduced after incubation with effector cells pre-treated with pembrolizumab, but that it was not observed for the effector cells without pembrolizumab pretreatment. These results confirmed that pembrolizumab served as a chicken PD-1 blocker and modulated the PD-1/PD-L1 pathway, therefore restoring the cytotoxicity of effector T cells. To strengthen the use of pembrolizumab *in ovo*, more analysis can be expected in future studies. For example, Surface Plasmon Resonance (SPR) binding analysis can be used to identify the chicken PD-1 epitopes binding to pembrolizumab. If the chicken PD-1 binding region for pembrolizumab overlaps with that of human PD-1, that could be the evidence needed to explain the mechanism of action of pembrolizumab in the PD-1/PD-L1 signaling pathway *in ovo*.

With these encouraging *in vitro* results, we continued the *in ovo* evaluation of pembrolizumab using the CAM assay. We first checked the sustained expression of PD-L1 on H460 tumors after *in ovo* xenografting, which is a critical factor for ensuring the PD-1 blocker’s performance. In Figure 5, the Western blot revelation confirmed that the CAM model is a suitable *in vivo* tumor cell xenografting model for IO study, keeping the PD-L1 immune epitope expression on tumor cells. Thus, the anti-tumoral performance of pembrolizumab was further evaluated *in ovo* and its tumor growth inhibition effect was confirmed (Figure 6). To better explain the mechanism of action of pembrolizumab *in ovo*, its performance was studied by evaluating the immune infiltration of tumors. The significant up-regulation observed with different immune markers, CD45, CD3, CD4, CD8, and CD56, revealed the evident immune cell infiltration in tumors (Figure 8), which was one more favorable proof of the PD-1/PD-L1 blockade as a main mechanism in the CAM assay, allowing the tumor to grow in this immune-competent model.

Treatment-related adverse events (or toxicity) are an essential consideration in drug development including IO drugs and must be evaluated in the preclinical phase. Although the immune checkpoint blockade provides important clinical benefits, it is frequently associated with a unique spectrum of side effects that are termed “immune-related adverse events” (irAEs), and sometimes may develop severe and life-threatening dysimmune toxicities [46,47]. Given these concerns, a quick and effective identification of the maximal tolerated dose (MTD) is critical for drug validation. The simplicity and cost-effectiveness of the *in ovo* model answers to this requirement well, for drugs of any class. We performed this pilot study for pembrolizumab (data not shown), through which we easily determined the doses of PD-1/PD-L1 inhibitors for *in ovo* efficacy testing. Indeed, pembrolizumab showed anti-tumor efficacy at both tested doses, without inducing any evident *in ovo* toxicity (Figure 7).

Lastly, to ensure the standard use of the chicken embryo model for PD-1 inhibitor studies, pembrolizumab was also tested on another MDA-MB-231 triple-negative breast cancer model, as well as another anti-human PD-1, nivolumab (OPDIVO^®^). Furthermore, as the PD-1/PD-L1 blockade could be achieved on either ligand, PD-1 or PD-L1, we further tested two anti-human PD-L1 agents, atezolizumab (TECENTRIQ^®^) and avelumab (BAVENCIO^®^), *in ovo*. The evident tumor growth regression was observed in all tests, at safe doses (Figure 9 and Figure 10). All these proofs strengthen the use of the chicken embryo model as an alternative *in vivo* model for studying PD-1/PD-L1 inhibitors. When discussing the challenge in using the chicken embryo model for IO research, some distinct differences between the human and the chicken immune systems should be taken into account [23]. Indeed, there exist some structural and functional differences between humans and chickens for other IO targets. For example, chickens are not able to produce IgG, but do secrete another immunoglobulin, IgY; mammalian IgG and avian IgY functions are equivalent, but the main difference resides in their structure [48,49]. Based on this fact, potential discrepancies in immune responses should be considered when using this model. To overcome this issue, genetic modifications could be a potential approach for making the chicken embryo model more suitable for IO study [50].

## 5. Conclusions

Even though they are different species, humans and chickens can build comparable immune responses to human tumors. The chicken model can be considered suitable for immune-based studies, due to its valuable contributions to our understanding of immunology [51]. A robust immune response can be induced in the chicken embryo model, even with a progressively mature immune system. This makes the CAM assay pertinent for immune-based studies, due to a physiological and efficient immune environment [23]. These characteristics allow recapitulating not only the immune system’s defense against tumor cells, but also the tumor immune escape via immune checkpoints. In this study, we applied the CAM assay to test PD-1/PD-L1 inhibitors. All the findings show the pertinence and the high potential for using this model to test more IO drugs on a large spectrum, and for validating IO combination regimens. Furthermore, many additional analyses can also be considered using the CAM assay, including the neo-angiogenesis analysis, tumor growth, metastatic invasion, transcriptomics, and many others. Moreover, the use of the chicken embryo model has shown many strong advantages over classical models, including the simplicity in egg management and handling, cost-effectiveness, time efficiency, and 3R compliance.

All these benefits illustrate that the chicken embryo model is a viable alternative *in vivo* model, which is fast and reliable for use in anti-cancer IO drug discovery.

## 6. Patents

Partial results reported in this manuscript have been used for the application of patent “WO2020089561A1”.

## Data Availability

Not applicable.

## Supplementary Materials

The following supporting information can be downloaded at: https://www.mdpi.com/article/10.3390/cancers14133095/s1, Figure S1: Western Blot Analysis of PD-L1 Expression on *in ovo* Xenografted H460 Tumors.
