# Supplementary material for: PD-1/PD-L1 Checkpoint Inhibitors Are Active in the Chicken Embryo Model and Show Antitumor Efficacy In Ovo
Source: Cancers (Basel). 2022 Jun 23;14(13):3095. doi: 10.3390/cancers14133095 (PMC9264844; doi:10.3390/cancers14133095)
Supplement: Supplementary file 1 [file cancers-14-03095-s001.zip › cancers-1774855-supplementary.pdf]

cancers

MDPI

Supplementary Material

PD-1/PD-L1 Checkpoint Inhibitors Are Active in the Chicken Embryo Model and Show Antitumor Efficacy *In Ovo*

Yan Wang [1,*], Xavier Rousset [1], Chloé Prunier [1], Paul Garcia [1,2,3], Emilien Dosda [1], Estelle Leplus [4] and Jean Viallet [1,2]

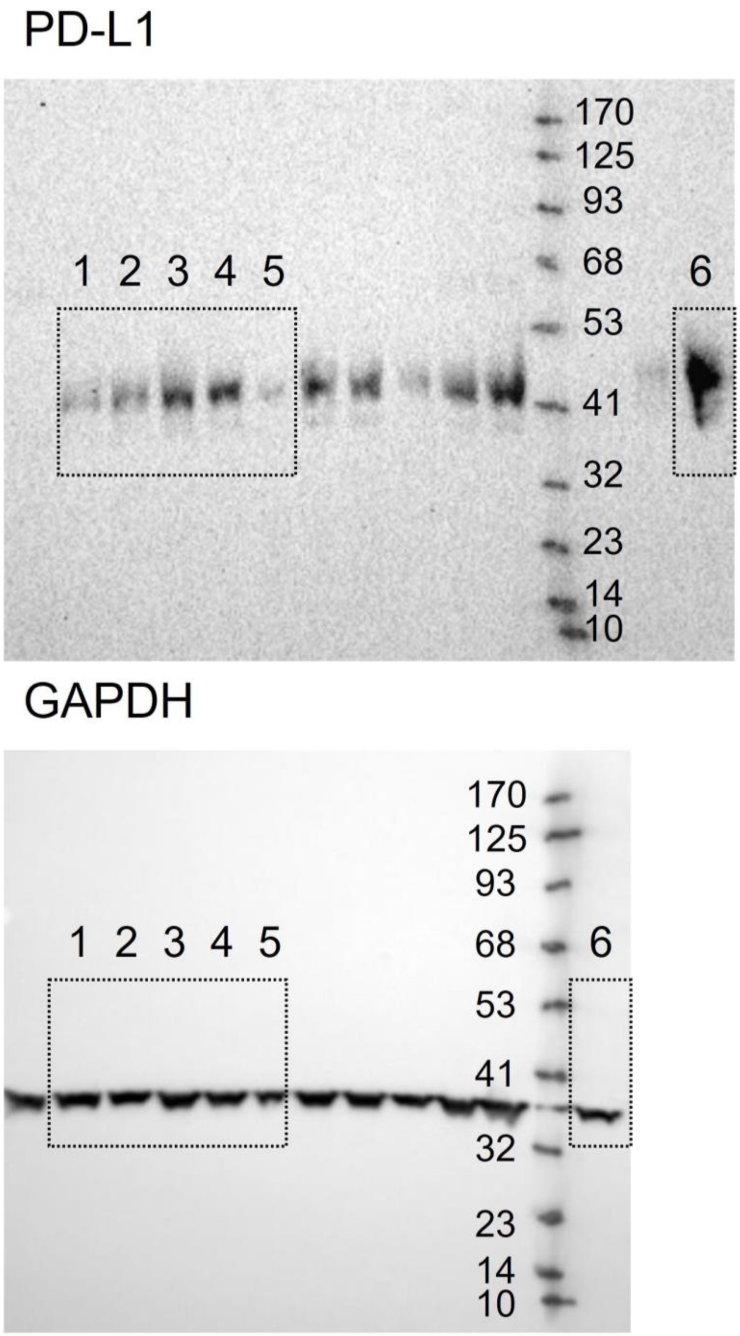

Figure S1. Western Blot Analysis of PD-L1 Expression on *in ovo* Xenografted H460 Tumors.
